# Supplementary material for: OsPMS1 Mutation Enhances Salt Tolerance by Suppressing ROS Accumulation, Maintaining Na+/K+ Homeostasis, and Promoting ABA Biosynthesis
Source: Genes (Basel). 2023 Aug 14;14(8):1621. doi: 10.3390/genes14081621 (PMC10454155; doi:10.3390/genes14081621)
Supplement: Supplementary file 1 [file genes-14-01621-s001.zip › genes-2497772-supplementary.pdf]

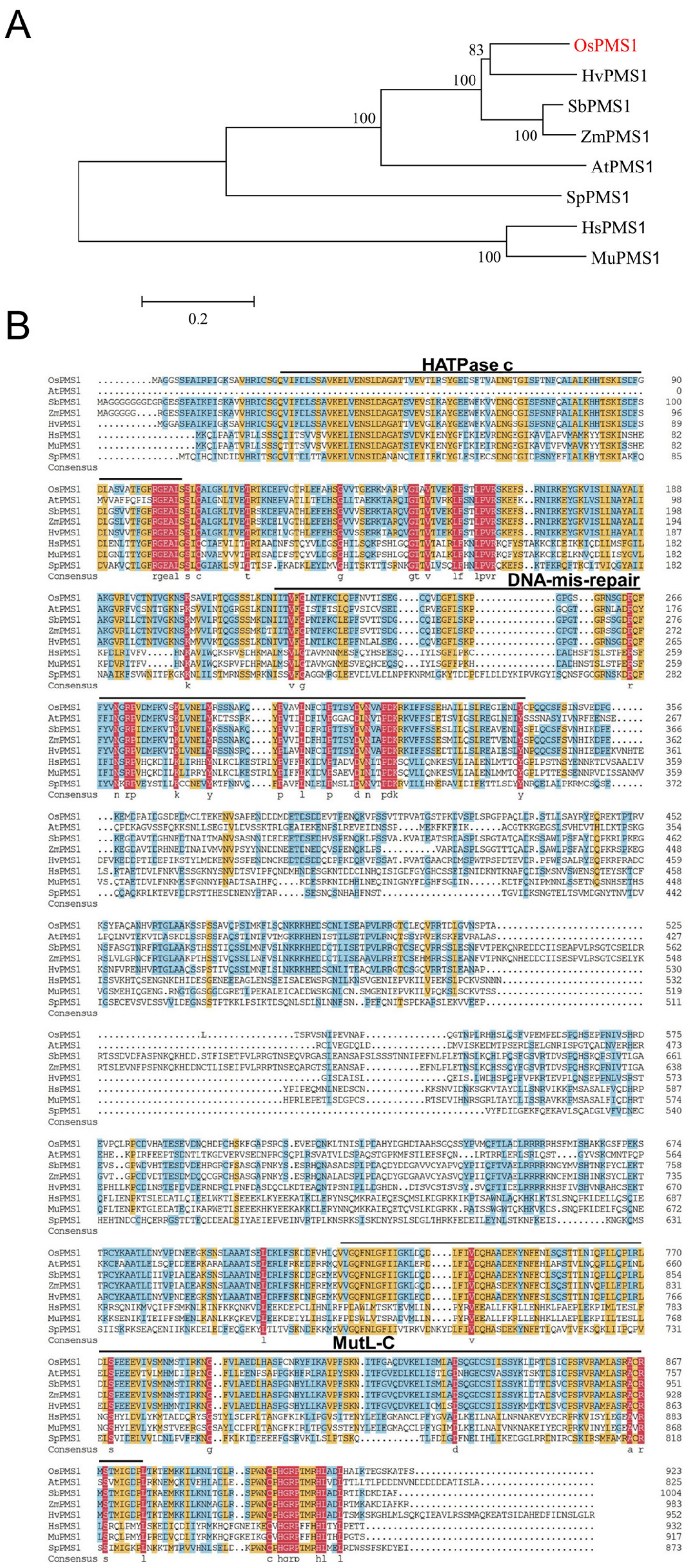

Figure S1. Evolutionary relationships and conserved structures of PMS1 homologous proteins. (**A**) Evolutionary relationships of PMS1 homologous proteins. (**B**) Conserved structures of PMS1 homologous proteins.OsPMS1, *Oryza sativa;* HvPMS1, *Hordeum vulgare*; SbPMS1, *Sorghum bicolor*; ZmPMS1, *Zea mays*; AtPMS1, *Arabidopsis thaliana*; SpPMS1, *Schizosaccharomyces pombe*; HsPMS1, *Homo sapiens*; MuPMS1, *Mus musculus*.

Table S1. Primers used for vector construction in the study.

Name	Sequence (5'-3')
q *OsPMS1* F	GCCAGAAGATTCACCACAGC
q *OsPMS1* R	TGAACATCGACTGGGAGCAC
OsActin F	GTGGTCGCCCCTCCTGAAAG
OsActin R	GGCTTAGCATTCTTGGGTCCG
LOC-*OsPMS1*-BamHI F	gctctagaactagtggatccATGGCCGGCGGCTCGTCGCC
LOC-*OsPMS1*-Sa1I R	ggccccccctcgaggtcgacTGAGAAAGTTGCCTTCGATC
OsPMS1-gRNA F1	GGCACGGCCGATCGGCAAGTCGG
OsPMS1-gRNA R1	AAACCCGACTTGCCGATCGGCCG
OsPMS1-gRNA F2	GCCGGAAGCTGACGGTCGAGACG
OsPMS1-gRNA R2	AAACCGTCTCGACCGTCAGCTTC
q*OsbZIP23* F	TCGCGCCAGAGGAAACAG
q*OsbZIP23* R	GGTCCAACTTGTCGGCTCAT
q*OsSAPK6* F	GAGCTCGTCGCCATGAAGTA
q*OsSAPK6* R	TGAGCACGACCTCCTTGAAC
q*OsNCED4* F	CACGGCGGAGAAGTTCATCT
q*OsNCED4* R	GTCCACCACCACCAGCTC
q*OsbZIP66* F	ACAGGGAGTCAGCAGCTAGA
q*OsbZIP66* R	TGACCGCCTCTAATACCTGA
q*OsDHAR1* F	CTCGGCGACTGTCCATTCTC
q*OsDHAR1* R	TCTTCAGAAACCAGTCGGGC
q*OsTZF1* F	CCTTGGGAGTTTCCACCTCG
q*OsTZF1* R	AGGGGAGAAGGGTAGAAGGC
q*SIT1* F	CCTTGGGAGTTTCCACCTCG
q*SIT1* R	AGGGGAGAAGGGTAGAAGGC
q*OsHAK5* F	ACTGCACATGGAAGCTGGAA
q*OsHAK5* R	TGGTGTCCTTGATCCCGTTG
q*OsLEA3-2* F	CAAGGACGCCACCAAGGAC
q*OsLEA3-2* R	CTTCTCCTTGATCGCCCCC

Table S2. Effects of NaCl treatment on root length, fresh weight, and dry weight of rice.

NaCl concentration mmol/L	Lines	Root length /cm	Fresh weight of plantlet /g	Dry weight of plantlet /g
0	WT	10.03±0.21	0.276±0.020	0.048±0.023
	ospms1-1	7.93±0.15**	0.282±0.008	0.052±0.015
	ospms1-2	9.83±0.25	0.285±0.012	0.051±0.021
150	WT	8.63±0.15	0.130±0.002	0.039±0.002
	ospms1-1	7.86±0.49	0.136±0.003*	0.054±0.001**
	ospms1-2	8.93±0.15	0.152±0.001**	0.056±0.002**

Results are the mean ± SD of three replicates. (*$p < 0.05$ and **$p < 0.01$, Students' t test).

Table S3. Statistical table of RNA-seq data.

Samples	Clean reads	Clean bases	GC (%)	Q30 (%)
NWT-1	51049662	7634470994	55.86	93.15
NWT-2	54324576	8120621426	55.55	92.66
NWT-3	47164610	7046387112	54.82	93.41
SWT-1	45152272	6746030816	54.06	93.03
SWT-2	53659752	8020084484	54.18	93.04
SWT-3	67929570	10157992272	53.94	93.34
NP1-1	48468220	7244333228	55.52	92.96
NP1-2	58616538	8760778922	54.62	93.47
NP1-3	47109158	7042816240	54.96	93.02
SP1-1	48673430	7264046438	53.73	93.04
SP1-2	47671274	7117573258	53.63	93.32
SP1-3	50173952	7502564544	53.19	92.34

NWT: wild-type before salt stress; SWT: wild-type after salt stress; NP1:*ospms1* mutant before salt stress; SP1: *ospms1* mutant after salt stress.
